# Increased risk of postpartum hemorrhage in women with endometriosis and adenomyosis: a prospective cohort study

**DOI:** 10.3389/fendo.2026.1718224

**Published:** 2026-06-30

**Authors:** Teresa Mira Gruber, Greta Ebeling, Wolfgang Henrich, Sylvia Mechsner

**Affiliations:** 1 Department of Obstetrics, Charité – Universitätsmedizin Berlin, Corporate Member of Freie Universität Berlin and Humboldt Universität zu Berlin, Berlin, Germany; 2 Endometriosis Centre, Charité – Universitätsmedizin Berlin, Corporate Member of Freie Universität Berlin and Humboldt Universität zu Berlin, Berlin, Germany

**Keywords:** adenomyosis, endometriosis, PPH, pregnancy, risk factors

## Abstract

**Introduction:**

A growing body of evidence suggests that women with endometriosis and adenomyosis are at an increased risk of complications during pregnancy and childbirth. Many of these complications are associated with increased blood loss. This study aims to evaluate and quantify the risk of postpartum hemorrhage in women with these conditions.

**Methods:**

We conducted a prospective cohort study of women with endometriosis and adenomyosis, analyzing their peripartal blood loss. Matching was performed for age and mode of conception. We defined postpartum hemorrhage as blood loss of ≥1000 mL, regardless of mode of delivery, according to the ACOG definition.

**Results:**

In our study, we examined 82 women with endometriosis and adenomyosis and 82 women without. A total of 11 women in the study group experienced postpartum hemorrhage, which is significantly higher than the rate in the control group (13% vs 1%). The unadjusted odds ratio for postpartum hemorrhage in the EM group was 8.74 (CI:2.01-82.00). The p-value was 0.002. In the adjusted analysis, participants with EM had a significantly increased risk of PPH compared to those without EM (OR:7.56, 95% CI:1.39-83.70, p=0.018), while gravidity and parity had no significant effect on the risk of PPH in our cohort.

**Conclusion:**

Our study found that women with endometriosis and adenomyosis were significantly more likely to experience postpartum hemorrhage than women without these conditions. These findings should be interpreted with caution, given the small sample size and wide confidence intervals. However, they suggest that women with endometriosis and adenomyosis may benefit from giving birth at hospitals with experience in managing postpartum hemorrhage.

## Introduction

Endometriosis (EM) is a common gynecological condition, affecting approximately one in five premenopausal women (1). Concomitant adenomyosis (AM) is present in up to 90% of cases (2). The influence of EM and AM on pregnancy and its outcome remains to be elucidated, but a growing body of evidence suggests that pregnant women with EM and AM may experience complications during late pregnancy and childbirth (3). In early pregnancy, EM has been linked to an increased risk of miscarriage and ectopic pregnancy. Later in pregnancy, an increased risk of preterm birth and premature rupture of the membranes, in addition to an increased risk of having a low-birthweight infant or developing hypertensive pregnancy disorders (including preeclampsia), has been described. Women with EM/AM are also at an increased risk of placental disorders, including placenta previa (PP) (4–6). There is evidence that women with EM may experience increased blood loss during cesarean delivery (CD) (7). An indirect marker of postpartum hemorrhage (PPH), namely, an increased rate of postpartum hysterectomy, has also been observed in women with EM (8). However, evidence on PPH in women with EM/AM is limited and contradictory (9).

PPH is the leading cause of maternal morbidity and mortality (10). To improve clinical management, risk stratification is crucial. Therefore, we conducted the following prospective cohort study to assess the risk of PPH in women with EM/AM compared to those without the diagnosis. We hypothesized that women with EM and AM have a significantly increased risk of postpartum hemorrhage compared with women without these conditions.

## Methods

The study methods are described in accordance with the Strengthening the Reporting of Observational Studies in Epidemiology (STROBE) guidelines (11).

### Study design

This prospective cohort study involved following a group of pregnant women with EM/AM during childbirth and comparing them with a control group without these conditions. We present an exploratory secondary analysis from an ongoing parent study. Peripartum blood loss was defined as a secondary outcome in the study protocol. The study received ethical approval from the Charité Ethics Committee (EA1/294/21).

### Cohort

From January 2022, pregnant women with a history of EM or AM were recruited at the Department of Obstetrics, Charité – Universitätsmedizin Berlin. We followed the definition of EM proposed by Keckstein et al. as an inflammatory disease characterized by the presence of endometrium-like epithelium, with or without accompanying stromal cells outside the endometrium and myometrium. AM was defined accordingly as the presence of ectopic endometrial tissue with glands and stromal cells infiltrating the myometrium (12). EM was classified using the revised American Society for Reproductive Medicine (rASRM) score for pelvic EM (13). The #ENZIAN classification was used to describe all forms of EM and AM, with special regard to deep infiltrating EM (DIE) (12). The diagnosis was self-reported. The implemented questionnaire was reviewed in a preliminary study by our group (6). The questionnaires collected information on patients’ medical histories, and participants were motivated to present clinical records and surgery reports to substantiate self-reported data. Nevertheless, we acknowledge that self-reported data my lead to misclassification and may substantially affect the findings. Clinical data concerning pregnancy and childbirth were extracted from the clinical software ViewPoint, version 6.

### Inclusion criteria

All participants were at least 18 years of age and able to give informed consent. They were included in the study before giving birth.

### Exclusion criteria

Women with a history of cesarean delivery (CD) or other cavity-opening operations were excluded. The study excluded cases of multiple pregnancies, severe maternal diseases, and fetal malformations.

### Control group

For each pregnant woman in the EM group, we found a matched individual based on age and mode of conception. The control pool consisted of all women without EM/AM who gave birth between 2019 and 2023 at Charité. From this pool, we randomly assigned one matching individual to each case.

### Measurement of blood loss

We documented the cumulative blood loss during delivery. PPH was defined as blood loss of at least 1000 mL during childbirth, regardless of the mode of delivery, according to ACOG guidelines (14). Blood loss was assessed using routine clinical methods. In our clinic, a quantitative blood collection bag is used to objectively quantify patients’ blood loss. The bag is used when visual blood loss exceeds the “normal” limit. For vaginal and operative vaginal deliveries, it is applied when blood loss exceeds 250 mL. During a cesarean section, we use a surgical suction device. For extensive blood loss from various sources (e.g., the blood collection bag and surgical swabs), we use the gravity method. These measurement methods are standardized for all deliveries, and staff are trained in PPH management. Transfusion criteria are strict, and blood transfusion is only considered in symptomatic patients with a Hb<7 g/dL.

### Bias

The Charité obstetrics department is a level one perinatal clinic and a tertiary care center. Accordingly, it treats more pregnant women with high-risk pregnancies than other centers.

Although tools to measure blood loss are part of the clinical routine, estimates are prone to underestimation (15). We furthermore acknowledge that our clinical routine in estimating blood loss may introduce ascertainment bias.

As the recruitment period for the control group overlaps but is not identical to that of the recruitment period of the EM group (2019–2023 vs. 2022–2024, respectively), a time-related bias is possible.

### Statistical methods

We compared pregnancy histories between the study groups. For categorical outcomes, we used Pearson’s chi-square test (X2) when each group included at least five cases for the variable. If any group had fewer than five cases, Fisher’s exact test was applied, and odds ratios (ORs) and 95% confidence interval (CIs) were calculated. To compare parity and gravidity in the study groups, we performed a non-parametrical Wilcoxon-rank-sum-test (WRST).

To assess the association between PPH and the exposure variables, we performed univariable and multivariable Firth’s penalized logistic regressions and calculated ORs with 95% CIs. The multivariable model included gravidity (primigravida vs. non-primigravida), parity (primipara vs. non-primipara), and fibroid and EM status (yes vs. no). To assess potential multicollinearity among the predictors (EM, parity, and gravidity), we calculated the variance inflation factor (VIF) and accepted values below a threshold of five. We further divided the study group by EM and fibroid statuses and performed an adjusted penalized logistic regression for each possible combination. To compare the absolute blood loss in the study groups, we performed the WRST, calculated interquartile ranges (IQR) and medians, and visualized the results in a boxplot. We further stratified PPH risk in women with EM by mode of delivery and calculated the risk for PPH for EM subgroups (AM, DIE, and AM+DIE). To compare hemoglobin (Hb) values, we used a two-sided t-test. All analyses were conducted using R (version 4.4.1).

## Results

In our study, we included 82 women with EM and 82 women without EM. A total of 11 births in the EM group resulted in PPH (≥1000 mL blood loss), compared with 1 birth in the control group (13% vs. 1%) (**Table 1**). The odds ratio (OR) for PPH in the EM group was 12.55 (95% confidence interval (CI): 2.35–232.23). The p-value was 0.017.

**Table 1:** Stratification of participants into primigravida and primipara vs. non- primigravida and non-primipara according to their EM status and the occurrence of PPH.

EM/AM status and PPH	Primigravida, n = 75 (%)	Non-primigravida, n = 89 (%)	Primipara, n = 115 (%)	Non-primipara, n = 49 (%)
EM	44 (59)	38 (43)	68 (59)	14 (29)
No EM	31 (41)	47 (57)	47 (41)	35 (71)
PPH (≥1000 ml)	9 (12)	3 (3)	11 (10)	1 (2)

### Study group selection

**Figure 1:**
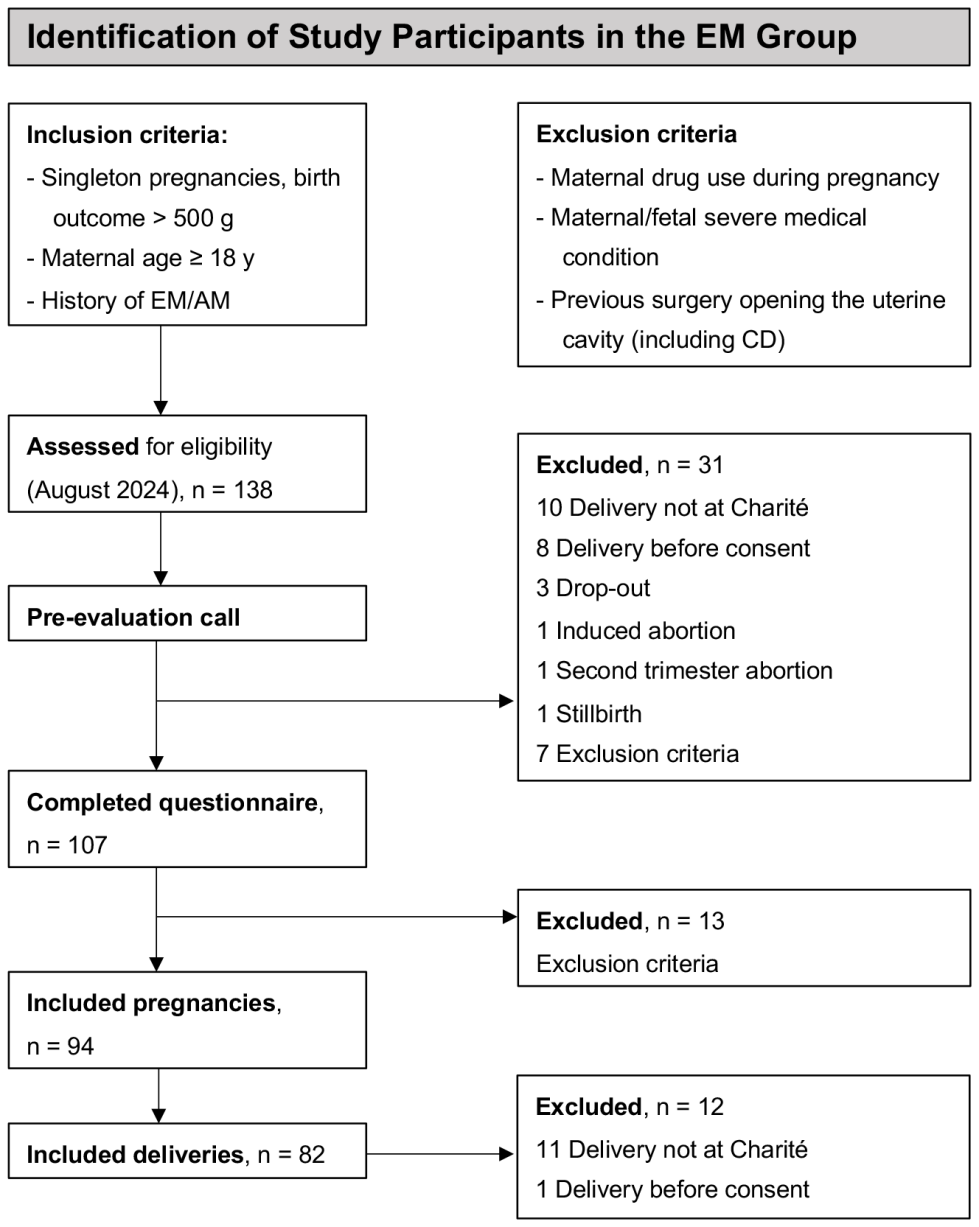
Study flow diagram according to Strobe guidelines.

Applying the inclusion criteria, 138 patients were initially eligible for the EM group. During pre-evaluation telephone screening, we excluded 10 patients who planned delivery outside Charité, 8 patients who delivered before providing consent, 3 dropouts, 1 patient who had an induced abortion, 1 patient with a second-trimester abortion, 1 patient with a stillbirth, and 7 patients who met exclusion criteria. The remaining 107 participants completed questionnaires, which led to further exclusion of 13 patients who reported exclusion criteria. An additional 11 patients delivered outside Charité, and 1 patient completed the questionnaire but never handed in her consent. Finally, 82 participants with EM were included in the study (see **Figure 1**).

### Cohort description

The median age of women at the time of delivery was 34 years in both groups, and 23 (28%) pregnancies in each group were achieved via IVF/ICSI. In the EM group, 43 (52%) women were primigravida compared with 31 (38%) in the control group. Primipara accounted for 68 (83%) in the EM group and 47 (57%) in the control group. In the EM group, 19 patients (23%) reported fibroids, whereas 3 patients (4%) in the control group had diagnosed fibroids. In the EM group, 1 patient was obese (body mass index (BMI) over 30) (1%) compared with 9 patients in the control group (11%). In the EM group, labor was induced in 19 cases (23%) compared with 21 labor inductions in the control group (26%) (see **Table 2**).

**Table 2:** Characteristics of study participants in the cohort and control groups according to risk factors for PPH.

Characteristics	EM	Controls
Age (min.–max.)	34 y (21–47 y)	34 y (21–47 y)
	n = 82 (%)	n = 82 (%)
IVF/ICSI	23 (28)	23 (28)
Primigravida	43 (52)	31 (38)
Primipara	68 (83)	47 (57)
Fibroids	19 (23)	3 (4)
Obesity (BMI > 30)	1 (1)	9 (11)
Labor induction	19 (23)	21 (26)

When comparing the distribution of the number of pregnancies and births between the study groups, statistically significant differences were observed (Gravida: p = 0.007; Para: p < 0.001). To account for these differences, we further included both variables as covariates in the adjusted analysis (see **Table 3**).

**Table 3:** Absolute number of pregnancies and births in cohort and control groups.

Pregnancies and Births	EM, median (min.–max.)	Controls, median (min.–max.)	P-value
Gravida	1 (1 – 7)	2 (1 – 8)	0.007*
Para	1 (1 – 2)	1 (1 – 6)	<0.001*

*Asymptotic significance (Wilcoxon rank sum test).

### Women with EM and AM

A total of 67 participants with EM had already undergone surgery due to their diagnosis prior to the index pregnancy (82%). Only 25 participants stated that they were connected to an EM center (30%). A total of 34 participants reported DIE (41%), and 38 participants declared that they were affected by AM (46%). In all, 45 pregnant women with EM provided a classification according to rASRM, and 56 according to #ENIZIAN (12) (see **Table 4**).

**Table 4:** EM classification of participants according to rASRM and #ENZIAN.

Classification	n = 82 (%)
DIE	34 (41)
AM	38 (46)
#ENZIAN	n = 56 (%)
Peritoneum	31 (53)
Endometrioma	23 (41)
DIE	20 (36)
AM	23 (41)
rASRM	n = 45 (%)
Stadium I	19 (42)
Stadium II	9 (20)
Stadium III	5 (11)
Stadium IV	12 (27)
Without classification	15 (18)

### Previous pregnancies

Seven participants in the EM group had an ectopic pregnancy compared to one participant in the control group (18% vs. 2%, OR: 10.37, 95% CI:1.24–486.09, p = 0.020). There was one stillbirth in the EM group (3%). A miscarriage was reported by 23 participants in the EM group and 25 in the control group (58% vs. 49%, X^2^: 0.35, p = 0.553), and three pregnant women with EM reported a history of termination of pregnancy (TOP) compared to five in the control group (8% vs. 10%, p = 1.000) (see **Table 5**).

**Table 5:** Comparison of previous pregnancies in the cohort and control groups.

Pregnancy outcome	EM, n = 40 (%)	Controls, n = 51 (%)	OR/X^2^	95%CI	P-value
Ectopic pregnancy	7 (18)	1 (2)	OR: 10.37	1.24 – 486.09	0.020*
Intrauterine death	1 (3)	–	–	–	–
Miscarriage	23 (58)	25 (49)	X^2^: 0.35	–	0.553
Termination of pregnancy	3 (8)	5 (10)	OR: 0.75	0.11 – 4.14	1
n. s.	–	4 (8)	-	–	–

### Mode of delivery

In all, 35 women with EM and 55 without gave birth spontaneously (43% vs. 67%). A total of 14 participants from the EM group and 6 from the control group gave birth by vacuum extraction (VE) (17% vs. 7%). A CD was performed in 33 pregnant women with EM and 21 pregnant women from the control group (40% vs. 26%), including 13 vs. 8 primary (16% vs. 10%) and 20 vs. 13 emergency CDs (24% vs. 16%). To compare outcomes within birth mode groups, we stratified the analysis by birth mode (see **Table 6**).

**Table 6:** Risk for PPH stratified by birth mode.

Mode of birth	OR (EM vs. no EM)	95% CI	P-value
Vaginal birth	4.74	0.25–699.71	0.303
Vacuum extraction	1.67	0.08–255.90	0.755
Primary CD	2.37	0.34–27.51	0.399
Emergency CD	9.58	0.94–1301.59	0.058

### Placental site distribution in women with PPH

Of all 82 participants with EM, 35 had a placenta located at the posterior uterine wall (43%), 33 had a placenta located at the anterior uterine wall (40%), 7 had a fundal placenta (9%), and 7 had a placenta located at a side wall (9%). Of all 11 participants with EM and PPH, 8 had a placenta located at the posterior uterine wall (73%), 2 had a placenta at the anterior uterine wall (18%), and 1 had a fundal placenta (9%). In the control-group, one case of PPH was recorded with a placenta located at the anterior uterine wall.

### PPH in women with EM and AM

In 11 women with EM, the cumulative blood loss during delivery was between 1000 mL and 1500 mL (13% vs. 1%, OR: 8.74, 95% CI: 2.01–82.00, p = 0.002).

#### Adjusted analysis of risk factors

As pregnancy and birth rates differed between groups, we performed Firth’s penalized logistic regression and adjusted for gravidity and parity. In the adjusted analysis, participants with EM had a significantly increased risk of PPH compared to those without EM (OR: 7.56, 95% CI: 1.39–83.70, p = 0.018). In contrast, primigravida-status was not a significant risk factor for PPH (OR: 0.56, 95% CI: 0.52–6.87, p = 0.369), nor was primipara status (OR: 1.05, 95% CI: 0.17–5.84, p = 0.953). As all VIF values were below 5, no concerning multicollinearity was detected in the model (see **Table 7**).

**Table 7:** Risk for PPH adjusted for gravidity and parity.

Characteristics	PPH (% from group)	OR	95% CI	P-value
EM, unadjusted, n=82	11 (13)	8.74	2.01 – 82.00	0.002*
EM, adjusted, n=82	11 (13)	7.56	1.39 – 83.70	0.018*
Primigravida, n=75	9 (12)	0.56	0.52 – 6.87	0.369
Primipara, n=115	11 (10)	1.05	0.17 – 5.84	0.953

*significant at p < 0.05. As all VIF values were below 5, they indicated no concerning multicollinearity in the model.

We further evaluated potential modification of the PPH risk by EM and fibroid status, using participants without EM or fibroids as the reference group. Due to the low number of events, these analyses are clearly exploratory and not robust. When EM and fibroids coexisted, participants appeared to have the highest risk for PPH, with an OR of 58.02 (95% CI: 6.06–7733.00, p < 0.001). Women with EM but no fibroids had a lower but still increased risk for PPH, with an OR of 17.91 (95% CI: 2.05–2367.00, p = 0.005) (see **Table 8**). To substantiate our data, future research is needed.

**Table 8:** Risk for PPH adjusted for fibroids.

EM and fibroid status	PPH (% from group)	OR	95% CI	P-value
No EM nor fibroid, n=79	0 (0)	Reference		
EM and fibroid, n=19	5 (26)	58.02	6.06–7733.00	<0.001*
EM no fibroid, n=63	6 (19)	17.91	2.05–2367.00	0.005*

*significant at p < 0.05.

#### Risk for PPH stratified by birth mode

We further evaluated the effect of EM and AM on the risk of PPH, and stratified by birth mode. We acknowledge that the low event count may affect model stability. There was a non-significant tendency for a higher risk for PPH in emergency CDs in women with EM, with an OR of 9.58 (95% CI: 0.94–1301.59, p = 0.058). In all other birth modes, the ORs for EM were greater than 1 but showed no significance or trend: the OR for PPH with EM in vaginal births was 4.74 (95% CI: 0.25–699.7, p = 0.303), for VEs the OR was 1.67 (95% CI: 0.08–255.90, p = 0.755), and for primary CDs the OR was 2.37 (95% CI: 0.34–27.51, p = 0.399) (see **Table 6**).

#### Risk of PPH by EM subgroup

When divided into EM subgroups, no significant risk could be calculated for any subgroup. Within the 82 participants with EM, 20 had an AM diagnosis, 16 had DIE, 19 had both, and 27 had EM without AM or DIE. In the AM-only-group (n = 20), two cases of PPH occurred (10%). Compared to the EM-only group, the OR for PPH in the AM-only group was 0.95 (10% vs. 11%, 95% CI: 0.15–5.39, p = 0.950). In the DIE-only-group (n = 16), three cases of PPH were recorded (19%), and the OR was 1.82 (95% CI: 0.34–9.74, p = 0.473). In the group of participants with both AM and DIE (n = 19), three births were complicated by PPH (16%). Compared to the EM-only group, the OR was 1.49 (95% CI: 0.28–7.84, p = 0.630). None of these findings were statistically significant (see **Table 9**).

**Table 9:** Risk for PPH stratified by EM, AM, and DIE.

EM, n = 82	PPH (% from group)	OR	95% CI	P-value
EM, only n = 27	3 (11)	Reference		
AM, n = 20	2 (10)	0.95	0.15–5.39	0.950
DIE, n = 16	3 (19)	1.82	0.34–9.74	0.473
AM+DIE, n = 19	3 (16)	1.49	0.28–7.84	0.630

#### Absolute blood loss

**Figure 2:**
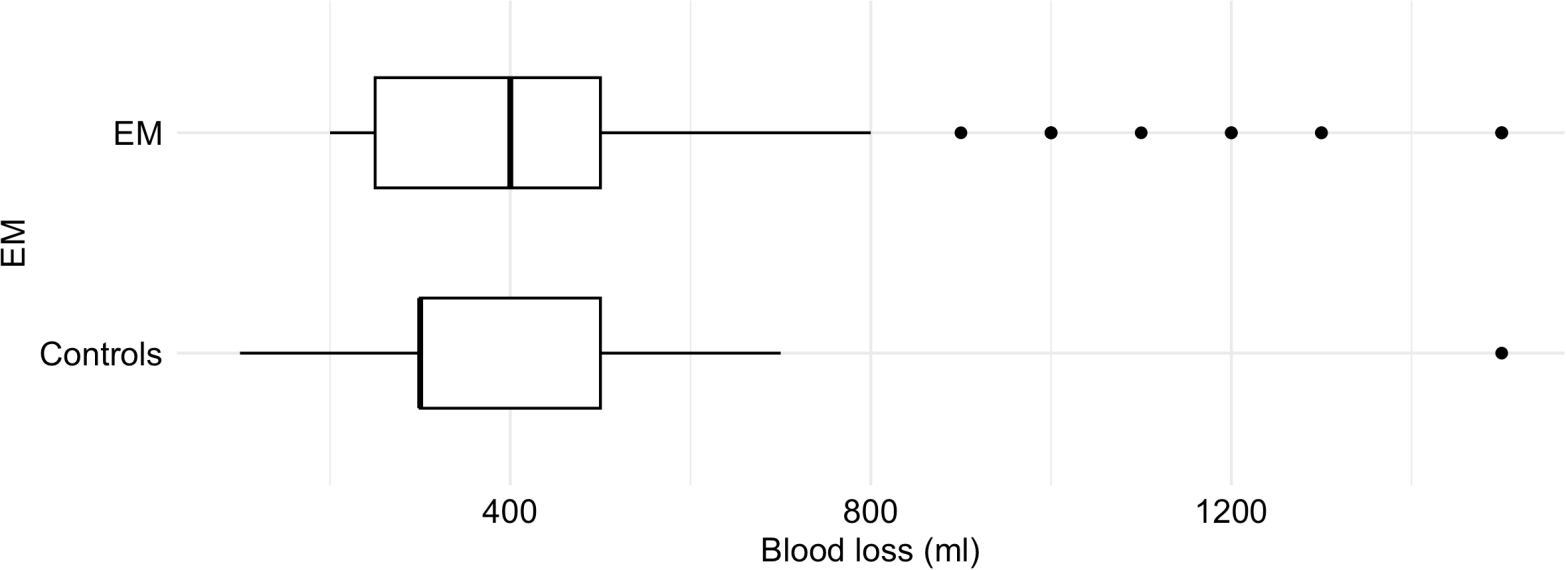
Boxplot of blood loss in the cohort and control groups.

The median blood loss in the EM group was 400 mL (IQR: 250–500mL) compared to 300 mL in the control group (IQR: 300–500mL). The p-value was 0.092, which indicates a tendency but no significant difference in median blood loss (see **Table 10**). In visualization, the EM group shows a greater range of blood loss and more cases of PPH (see **Figure 2**).

**Table 10:** Absolute blood loss in the cohort and control groups in mL.

EM status	Blood loss, median (mL)	Blood loss, IQR (mL)	P-value
EM, n = 82	400	300 – 500	0.092
Controls, n = 82	300	250 – 500	0.092

#### Hb levels in women with PPH

After delivery, women with PPH had a significantly lower Hb level than women in the control group. The mean was 8.7 g/dL compared to 11.0/dL in the control group (standard deviation (SD): 1.51; range: 6.6–10.7 g/dL). The p-value was < 0.001.

The mean Hb level in women with EM without PPH and in the control group did not differ significantly (p-value = 0.120). The mean was 10.5 g/dL (SD: 1.67; range: 6.6–15.5 g/dL) vs. 11.0 g/dl (SD: 1.60; range: 7–14.8). A total of 10 participants with EM and 3 participants without EM had no documented Hb value (12% vs. 4%). To calculate the Hb levels, we used only the available data; therefore, these results should be interpreted with caution.

#### Birth mode in women with PPH

Women with PPH gave birth via CD in nine cases: four primary and five emergency CDs. The reasons for primary CDs were fetal breech presentation (n = 2), vasa previa (n = 1), and PP (n = 1). Causes of increased blood loss were uterine atony, placenta accreta with placental bed bleeding, myometrial hypervascularization, and intraoperative tears.

In four cases, non-reassuring fetal status (NRFS) was the indication for emergency CD, and one was performed due to protracted labor.

Notably, in the group of NRFS, one uterine rupture during labor was detected and one spontaneous intra-abdominal hemorrhage occurred. In both cases, an emergency CD was performed, and both mother and child survived in good conditions.

In the context of vaginal delivery, two women experienced PPH. One was due to uterine atony following VE after prolonged labor, and one was due to placental remnants causing increased blood loss.

Notably, 8 out of 11 women presented with a placenta located on the posterior uterine or lower posterior uterine wall. One had placenta previa of the posterior uterine wall (details see **Table 11**).

**Table 11:** Detailed characteristics of women with PPH.

Patient number	Age	G/P	Mode of conception	Placenta localization	EM/AM classification	Weeks of gestation	Mode of delivery	Cause for the CD of VE	Cause for the bleeding	Uterotonics	Surgical procedures	Blood loss	Hb ante partum	Hb post-partum
1	36	1/1	IVF/ICSI	Lower posterior uterine segment	#ENZIAN Oo/2	37+0	Primary CD	Vasa previa	Placenta bed bleeding, atony	Sulprostone	Celox ® tamponade intrauterine	1000 mL	n. a.	10,6
2	36	2/1, 1x EU	Spontaneous	Posterior uterine segment	Adenomyosis	39+5	Primary CD	Mobile fetal presentation	Myometrial hypervascularization	Carbetocin, tranexamic acid	Celox® tamponade intrauterine, topical hemostatic sponge	1500 mL	12,1	9,6
3	48	1/1	IVF/ICSI	Posterior uterine segment	rASRM IV, adenomyosis, recto cervical EM, #ENZIAN P3, Oo/1, AB 2-3, C2, Peritoneum	39+0	Primary CD	Placenta previa	Placenta bed bleeding, atony	Oxytocin, tranexamic acid	Ligation	1300 mL	11,6	7,1
4	32	1/1	Spontaneous	Posterior uterine segment	rASRM I, Endometrioma uterus myomatosus	38+6	Prim CD	Breech presentation	Stony	Carbetocin, tranexamic acid	-	1300 mL	11,0	9,8
5	37	1/1	Spontaneous	Fundus	ENZIAN B1, rASRM II, uterus myomatosus	36+4	Secondary CD	FGR and NRFS	Placenta adherence	Carbetocinn, tranexamic acid	Manual placental removal, ligation	1000 mL	12,2	10,3
6	30	1/1	Stimulation of ovulation	Posterior uterine segment	rASRM IV, adenomyosis, uterus myomatosus	24+1	Secondary CD	NRFS	Spontaneous hematoperitoneum	Carbetocin, tranexamic acid	Ligation	1200 mL	10,5	6,9
7	35	2/1, partial mole pregnancy (2xcurettage)	IVF/ICSI	Anterior uterine segment	#ENZIAN: P1, uterus myomatosus	39+2	Secondary CD	Prolonged labor	Atony	Carbetocin	Topical hemostatic sponge	1000 mL	13,5	10,6
8	32	1/1	Spontaneous	Posterior uterine segment	Rectal EM	41+1	Secondary CD	NRFS, chorion-amnionitis	Myometrial hypervascularization, intraoperative tear	Carbetocin, tranexamic acid	Ligation and hemostatic powder	1500 mL	11,7	7,7
9	41	2/2, 1x vacuum extraction	Spontaneous	Posterior uterine segment	#ENZIAN P1, adenomyosis, uterus myomatosus	38+2	Secondary CD	NRFS	Uterine rupture	Carbetocin, Tranexamic acid	Uterine reconstruction	1500 mL	10,7	7,4
10	32	1/1	Spontaneous	Anterior uterine segment	#ENZIAN (s) P1, O2/0, T2/0, B0/2	41+4	Vacuum extraction	Prolonged labor	Atony	Misoprostol rectal	Atony	1200 mL	13,7	6,1
11	39	1/1	IVF/ICSI	Posterior uterine segment	Adenomyosis, rASRM IV	38+0	Spontaneous delivery	-	Placental remnants	Oxytocin and tranexamic acid	Manual placental removal	1100 mL	n. a.	9,0

#### Management of PPH

The management of PPH included manual placental removal, surgical ligation, and uterine repair. We further used intrauterine Celox^®^ tamponade and the application of topical hemostatic sponges. Moreover, the patients received tranexamic acid, misoprostol, carbetocin, and sulprostone. None of the women with PPH received red blood cell transfusion (details see **Table 11**).

## Discussion

### Principal findings

The rate of PPH in the EM/AM group was significantly higher than in the control group (13% versus 1%). The odds ratio for postpartum hemorrhage in the EM group was 8.74 (CI: 2.01–82.00). The p-value was 0.002. In the adjusted analysis, participants with EM had a significantly increased risk of PPH compared to those without EM (OR: 7.56, 95% CI: 1.39–83.70, p = 0.018), while gravidity and parity had no significant effect on the risk of PPH in our cohort. We excluded women who had undergone previous cesarean sections (CD) or other operations that opened the uterine cavity, performed matching of age and mode of conception, and adjusted for gravidity, parity, and fibroid status in order to eliminate major risk factors for PPH during delivery.

### Mechanisms of increased blood loss in women with EM/AM

We assessed the risk of PPH in women with EM and AM regardless of mode of delivery, according to the ACOG definition as a blood loss of at least 1000 mL during childbirth (14). Despite this, we analyzed the mode of delivery in relation to the risk of PPH (see **Table 6**). The results should be interpreted with caution due to the low event count. Nevertheless, the highest risk of PPH was detected in the emergency CD group. We consider as partly causative the known risk factors for PPH, including prolonged and protracted labor resulting in unscheduled CD (16, 17). These factors, in combination with EM- and AM-associated pathophysiological components contributing to increased blood loss during CD, may explain the high OR. In our study, mode of delivery may be considered both a risk factor and a confounder.

The following sections aim to frame hypotheses to explain the pathophysiology of increased risk of PPH in women with EM and AM:

#### Hormones and the arrest of labor

The literature describes the risk of PPH as particularly high in cases of vaginal operative birth and emergency CD due to their association with prolonged labor (16, 17).

#### Oxytocin receptor expression

The contractility of the uterus is mediated by oxytocin (OT) (18, 19). OT receptor (OTR) levels increase with gestational age and the onset of labor. However, when OT is used to induce and support labor, OTR levels decrease and the risk of PPH increases (20, 21).

During pregnancy, OTRs are expressed more abundantly in the fundus than in the lower uterine segment (22). Consequently, the contractility of the lower uterine segment is weaker than that of the corpus uteri after birth. If the placenta is then located in the lower part of the uterus, bleeding from the placenta may be observed due to uterine atony.

#### Myometrial contractility

Uterine motility is disturbed and described as hypermobility in women with EM/AM (23). Altered uterine mobility can lead to two consequences. First, the adenomyotic uterus may be less able to produce expulsive contractions, prolonging labor and increasing the risk of PPH. Second, postpartum uterine hypermobility may interfere with myometrial contractions to stop bleeding from the placental bed, increasing the risk of uterine atony. Additionally, architectural changes to the uterus in the advanced stages of the disease, such as abnormal fixation due to posterior uterine adhesions, thickening of the myometrium, and endometriotic lesions within it, may intensify the difficulty of achieving purposeful contractions (8, 24).

#### Placenta localization in women with EM/AM

In our cohort, we found that 8 of 11 women with PPH had a placenta located at the posterior uterine wall. One woman presented with posterior placental implantation, and one with a placenta at the lower uterine wall and vasa previa.

This is the first evidence that placentation and its localization may be influenced by EM/AM. An increased risk of PP in women with EM/AM has already been reported (6, 25), specifically an association between PPH from the posterior uterine wall and posterior extrauterine adhesions, which may be induced by AM and DIE (26). The increased vascularization of adenomyotic lesions, which are more prevalent in the posterior compartment in association with DIE, may provide a niche for placental growth.

Additionally, extrauterine adhesions may rupture and cause bleeding during CD if they are located outside the uterine cavity; therefore, exteriorization of the uterus should be avoided in women with AM/DIE.

#### Architectonical and inflammatory changes of soft tissue in women with EM and AM

One of our patients experienced spontaneous hemoperitoneum during pregnancy and delivered her baby at 24 weeks and 1 day of gestation via emergency cesarean section. Spontaneous hematoperitoneum in pregnancy may be triggered by extrauterine EM lesions in association with decidualization (27). The constant inflammatory environment in EM can lead to the formation of fibrotic tissue around EM lesions (28). These environmental conditions favor the formation of adhesions that, in the context of pregnancy, may rupture spontaneously during contractions or trauma.

We also observed one case of spontaneous uterine rupture during labor. While a hysterectomy is often required in such emergency situations, the mother and child in our case were in good condition after delivery, and we were able to repair the uterus. A tragic case described in the literature illustrates the underlying pathophysiology: a hysterectomy was performed due to a spontaneous uterine rupture during delivery, and a pathological examination of the uterus was conducted. On the side of the rupture, a complete absence of myometrial smooth muscle cells was observed, along with the presence of AM (29). The force of myometrial contractions during labor can cause the uterus to rupture in areas where there are adenomyotic changes to the myometrial wall.

#### Abnormal vascularization of the myometrium in women with EM and AM

In addition to the destabilizing effect of AM on the uterine architecture, the presence of endometrial-like tissue in the myometrium is associated with hypervascularization. Injury and repair mechanisms within the myometrium induce angiogenic factors, favoring myometrial hypervascularization and the development of unstable vessels. These changes have been described in the literature as the cause of abnormal uterine bleeding during menstruation in women with AM (30). During pregnancy and childbirth, uterine hypervascularization can lead to increased blood loss following miscarriage and in term pregnancies from the placental bed. We also observe increased bleeding from the uterine incision and uterine tears during CD.

### EM and concomitant AM in our cohort

In our cohort, AM was present in only 46% of patients, whereas a higher percentage would be expected. The majority of uterine factors discussed are directly associated with AM rather than ectopic EM. As only 30% of patients were seen in specialist EM units, the prevalence of AM may be underestimated in this group.

### Strength and limitations

This topic is clinically relevant in obstetrics and clinical medicine. Understanding the links between EM/AM and PPH improves antenatal risk stratification and peripartum management for mothers with EM/AM. This prospective study supports internal validity and standardized clinical assessment. Adjusted and unadjusted analyses of our primary endpoint showed an increased risk of PPH, suggesting a real difference despite the small number of patients. Nevertheless, we acknowledge limitations related to the low event count, possible residual confounding, and potential exposure misclassification. The multivariable analysis may be unstable, is exploratory, and the wide CIs calculated are explained by the small sample size. Matching was performed for age and mode of conception, as these were the greatest potential confounders in our cohort. To minimize the time period for control selection, matching for more variables was not feasible. We therefore performed a statistical analysis to adjust for other potential confounders, such as parity and mode of delivery. We acknowledge that our work is prone to selection bias and diagnostic misclassification, given that the history of EM/AM is self-reported. Temporal bias arising from the different recruitment periods of cases and controls may also have influenced our results; however, standard obstetric procedures within our clinic remained unchanged throughout the entire study period.

### Clinical implications

Our work may result in an increased awareness of PPH risk in primiparous women with EM/AM. To recognize placental disorders, specialized sonography during pregnancy can be performed. Furthermore, giving birth in a medical center with a blood bank and the capability to manage severe bleeding complications can reduce morbidity and mortality in women with severe PPH.

## Conclusion

In our study, women with EM/AM were found to be at significantly greater risk of postpartum hemorrhage than those without these conditions. Notably, the majority of the women with PPH in the EM/AM group were primipara.

However, these findings should be interpreted with caution given the relatively small number of events and wide confidence intervals. Nevertheless, they suggest that women with EM and AM may benefit from delivering at centers with experience in managing postpartum hemorrhage. Further research is needed to create more robust evidence.

## Data Availability

The raw data supporting the conclusions of this article will be made available by the authors, without undue reservation.
